# Pancreatic Steatosis and Survival Outcomes in Pancreatic Ductal Adenocarcinoma: A Systematic Review of the Current Evidence

**DOI:** 10.3390/diagnostics16152476

**Published:** 2026-08-06

**Authors:** Daniel Vasile Balaban, Manucu George, Mariana Jinga

**Affiliations:** 1 Faculty of Medicine, Internal Medicine and Gastroenterology Clinic, “Carol Davila” University of Medicine and Pharmacy, 020021 Bucharest, Romania; vasile.balaban@umfcd.ro (D.V.B.); mariana.jinga@umfcd.ro (M.J.); 2 Gastroenterology Department, Central Military Emergency University Hospital, 010242 Bucharest, Romania; 3 Academy of Romanian Scientists, Ilfov 3, 050044 Bucharest, Romania

**Keywords:** pancreatic steatosis, pancreatic ductal adenocarcinoma, prognosis, survival

## Abstract

**Background:** Pancreatic steatosis (PS) has emerged as a potential risk factor for pancreatic ductal adenocarcinoma (PDAC). While increasing evidence supports its role in pancreatic carcinogenesis, its prognostic significance after PDAC diagnosis remains unclear. We performed a systematic review to evaluate the association between PS and survival outcomes in patients with pancreatic cancer. **Methods:** A systematic search of PubMed and Scopus was conducted in May 2026 according to PRISMA guidelines. Observational studies assessing pancreatic steatosis by imaging or histology and reporting survival outcomes in PDAC patients were included. Risk of bias was evaluated using the Quality in Prognosis Studies (QUIPS) tool. **Results:** Five studies met inclusion criteria, comprising three imaging-based and two histology-based papers. Three studies reported findings suggestive of an adverse prognostic role of PS, whereas two found no significant association with survival. The strongest evidence originated from a study using quantitative histological assessment of pancreatic fat, which demonstrated that greater pancreatic fat accumulation was independently associated with poorer overall survival and recurrence-free survival following surgical resection. In contrast, imaging-based studies yielded inconsistent results. Histology-based studies consistently supported a negative prognostic impact, whereas CT-derived attenuation measures showed substantial variability. Risk of bias was moderate to high in most studies, largely due to retrospective designs, small sample sizes, heterogeneous definitions of pancreatic steatosis, and incomplete adjustment for metabolic confounders. **Conclusions:** Current evidence regarding the prognostic value of pancreatic steatosis in PDAC is limited and conflicting. While histology-based studies suggest an adverse impact on survival, imaging-based data remain inconsistent.

## 1. Introduction

Pancreatic ductal adenocarcinoma (PDAC), the most common histological subtype of pancreatic cancer, is one of the most lethal malignancies, with a prevalence almost equal to incidence. The dismal prognosis of PDAC, reflected by a 5-year survival rate barely reaching 13% [1], is caused by late-stage diagnosis, aggressive tumor biology with a chemoresistant immune-eluding tumor microenvironment and lack of screening programs.

**Figure 1:**
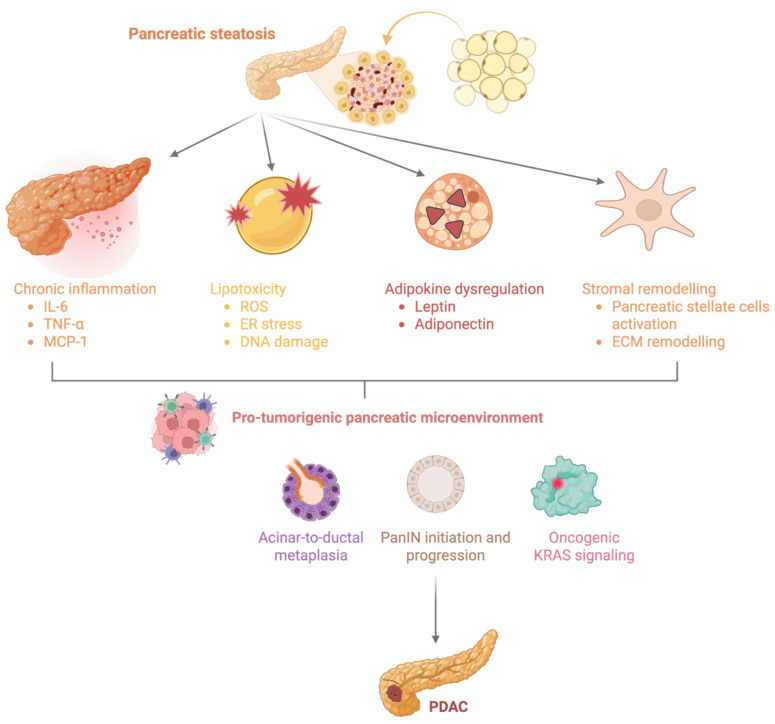
Proposed biological mechanisms linking pancreatic steatosis with PDAC development and progression (Created in BioRender. Balaban, D. V. (2026) https://BioRender.com/h1p4ppc).

Only 10% of PDAC cases are genetically driven, and the remaining majority develop sporadically, through the convergence of several risk factors. Among these factors, fatty pancreas (FP) has emerged as a significant contributor to PDAC occurrence [2,3,4]. Two meta-analyses have reported a high prevalence of pancreatic steatosis (PS) in patients with PDAC, with a six times higher probability of having PS among patients with pancreatic malignancy [5,6]. PS plays a key role in pancreatic carcinogenesis through multiple interconnected mechanisms, including chronic low-grade inflammation, adipokine dysregulation, lipotoxicity, and the creation of a pro-tumorigenic microenvironment. Excess intrapancreatic adipocytes secrete pro-inflammatory cytokines such as IL-6 and TNF-α and alter adipokine signaling through increased leptin and reduced adiponectin production, thereby fostering a chronic inflammatory microenvironment that promotes cellular proliferation, resistance to apoptosis, and neoplastic transformation [7,8,9]. Furthermore, pancreatic fat infiltration has been associated with acinar cell remodeling and an increased burden of pancreatic intraepithelial neoplasia (PanIN), while experimental studies have demonstrated that persistent acinar-to-ductal metaplasia (ADM) in the setting of oncogenic KRAS activation promotes progression to PanIN and ultimately pancreatic ductal adenocarcinoma [10,11], as shown in Figure 1. These observations support the concept that pancreatic steatosis is not merely an epiphenomenon of metabolic dysfunction but may actively contribute to PDAC initiation and progression.

**Figure 2:**
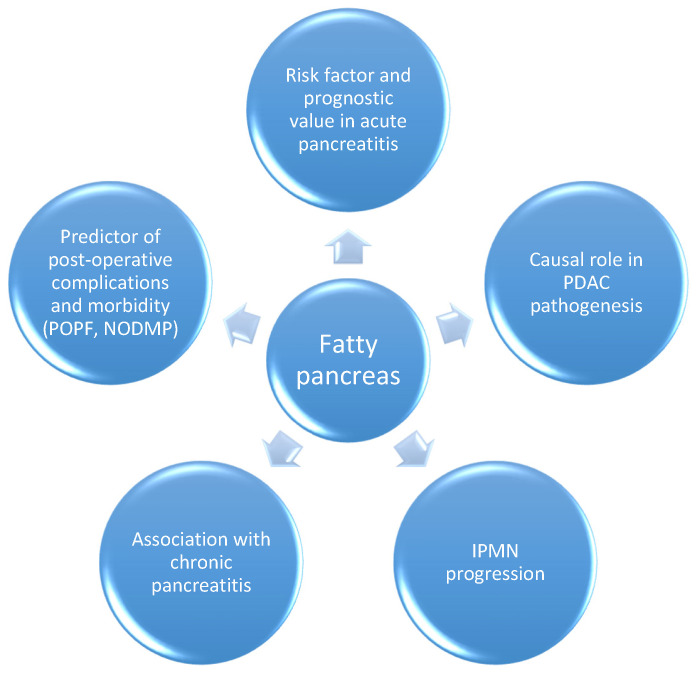
Fatty pancreas involvement and association with exocrine pancreatic diseases. Abbreviations: POPF—postoperative pancreatic fistula; NODMP—new-onset diabetes mellitus following pancreatectomy; PDAC—pancreatic ductal adenocarcinoma; IPMN—intraductal papillary mucinous neoplasm.

Beyond the association of PS as a causal factor for PDAC development [12], fatty infiltration of the pancreatic parenchyma has also been studied with respect to association with benign exocrine pancreatic diseases [13,14,15,16,17], surgical complications [18,19,20] or progression of precursor lesions [21]; see Figure 2. Moreover, FP has been reported as an independent adverse prognostic factor for survival in pancreatic neoplasia, as described in Gao et al.’s study on high-grade pancreatic neuroendocrine neoplasms [22].

Our aim was to analyze the current literature with regard to the prognostic role of FP in PDAC survival.

## 2. Methodology

We conducted a systematic literature search in two databases in May 2026, Pubmed and Scopus, using dedicated search strings (available as Supplementary File S1). The search criteria specifically targeted original observational studies (both cohorts and case–control studies) evaluating the prognostic significance of PS for PDAC survival. The search strategy included the core concepts “pancreatic steatosis,” “pancreatic adenocarcinoma” and “survival” together with their corresponding related terms. The framework behind by our review question was defined as: P (population)—PDAC patients, regardless of disease stage, treatment modality, or resectability status; E (exposure)—PS (fatty pancreas, pancreatic fat infiltration, non-alcoholic fatty pancreas disease, intrapancreatic fat deposition) assessed by imaging (CT, MRI, EUS), histopathology, or other validated methods; C (comparator)—patients without PS, or patients with lower pancreatic fat content compared with those with higher pancreatic fat content; O (Outcome)—survival outcomes, consisting of overall survival (OS), progression-free survival (PFS), recurrence-free survival (RFS), disease-free survival (DFS), cancer-specific mortality, and hazard ratios for mortality. This systematic review adhered to the updated PRISMA guidelines reported by Page et al. [23] (Supplementary File S3). The review was registered in the Inplasy study registry (INPLASY202660118).

Specifically, this review synthesizes evidence regarding pancreatic steatosis, as measured by imaging (computed tomography, magnetic resonance imaging) or histology, and its potential to serve as a reliable independent predictor for both overall and progression-free survival. We excluded studies referring to prognostic potential for survival of non-pancreatic adipose tissue distributions such as subcutaneous fat, visceral fat or other compartments. We also excluded studies evaluating only risk of developing PDAC or post-operative complications such as pancreatic fistula, or other outcomes, without reporting oncologic prognosis measures. Not least, we did not include case reports, small case series, conference abstracts or narrative reviews.

**Figure 3:**
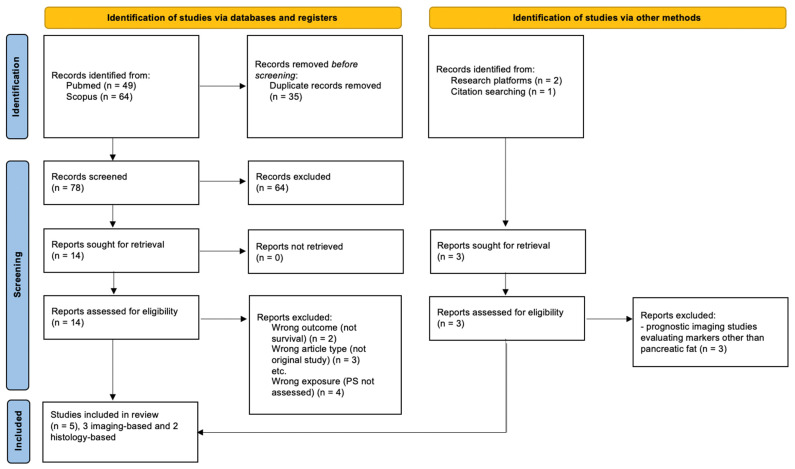
PRISMA flow diagram of the literature search, screening and study selection process.

Screening of titles and abstracts and selection of studies for final analysis were carried out by two examiners, with potential voting conflicts set to be decided by consensus decision after argumentative discussion. The flowchart of study search and selection is depicted in Figure 3. For each eligible study we extracted the following data: name of the first author and publication year, study design, sample size and population characteristics, fat assessment method, and survival outcomes. Data was extracted by one reviewer using a standardized form and independently verified by the second reviewer; disagreements were set to be resolved through discussion and consensus.

Risk-of-bias (RoB) assessment was done using the QUIPS tool [24], which evaluates six domains of potential bias in prognostic factor research: study participation, study attrition, prognostic factor measurement, outcome measurement, study confounding, and statistical analysis and reporting. Each domain was reported for all included studies as low, moderate or high risk of bias and presented as corresponding color—green (🟢), yellow (🟡) or red (🔴) bullet. RoB judgments were performed by one reviewer and checked by the second reviewer.

## 3. Results

Only five studies referring to survival outcomes were analyzed for the purpose of this review, which are summarized in Table 1. Among them, three reported findings suggestive of a poorer prognosis with PS, although the strength and directness of the survival association varied substantially (Mathur 2009; Mathur 2011; Fukuda 2025) [25,26,27], while the other two (Chan 2024; Dong 2025) [28,29] found no prognostic impact. Thus, the current evidence is conflicting, but there seems to be a trend toward a potential adverse prognostic role, particularly in studies using quantitative histologic assessment (Fukuda 2025 [27]) or CT-derived pancreatic attenuation (Mathur 2011 [26]). The strongest prognostic evidence came from Fukuda et al. 2025 [27], who demonstrated independent associations with both OS and RFS after multivariable adjustment.

The clinical setting and the available information on disease stage and resectability varied substantially across the included studies. Four studies evaluated exclusively surgically treated patients. The 2009 study by Mathur et al. [25] included 40 patients with resected pancreatic adenocarcinoma, equally divided between node-negative and node-positive disease; the full AJCC stage was not reported, while chemoradiation was reported in 83% and 94% of the two groups, respectively. In their 2011 paper, Mathur et al. [26] included 42 patients with pancreatic-head adenocarcinoma undergoing curative-intent pylorus-sparing pancreatoduodenectomy. Of these, 24 patients (57%) had nodal metastases, and all patients were reported to have completed gemcitabine-based adjuvant chemotherapy. Dong et al. [29] evaluated 116 patients undergoing radical surgery, including 48 with N1–2 disease. Fukuda et al. [27] included 87 consecutive patients undergoing potentially curative pancreatectomy; 74 patients (85.1%) had T3 disease, 48 (55.2%) had nodal involvement, and 62 (71.3%) achieved R0 resection. Neoadjuvant and adjuvant chemotherapy were administered in 12 patients (13.8%) and 57 patients (65.5%), respectively. In contrast, Chan et al. [28] included a mixed-stage population, with 53 of 101 patients (52.5%) having stage IV disease and only 26 (25.7%) undergoing surgery; details of systemic treatment were not reported. Overall, the available evidence predominantly originated from resected PDAC populations, while tumor-stage and treatment reporting remained incomplete and heterogeneous across studies.

In the first study looking at survival outcomes PDAC according to PS, Mathur et al. [25], reported a survival difference according to nodal status (18.9 in node-positive patients vs. 30.8 months in node-negative patients, *p* < 0.04), with increased pancreatic fat being associated with nodal metastases; inferentially, the authors concluded that PS promotes node dissemination and lethality of pancreatic malignancy. In a second landmark study involving CT-based assessment of PS, Mathur et al. [26] reported that lower PS is associated with dissemination and abbreviated survival, with lower attenuation of the pancreas seen in patients with lymphatic metastases (mean HU of 23 and 21 vs. 35 and 34 in pancreatic body and tail compared to node-negative patients), who demonstrated poorer survival. Critically, stratifying node-positive patients by pancreatic steatosis alone (body HU <30 vs. ≥30) did not reach statistical significance for survival, though there was a trend toward decreased OS with greater steatosis, suggesting pancreatic parenchymal fat may need the context of lymph node metastasis to exert its prognostic impact, or that the study was underpowered for this subanalysis. The other two studies using imaging-based assessment of PS provided contrasting results—while Chan et al. [28] found possible higher survival in patients with PS (30% vs. 14.6%, *p* = 0.322), Dong et al. [29] reported a worse prognosis in PS patients (19.5 months vs. 24.6 months, *p* > 0.05), and both lacked statistical significances. The trends in survival seen in PDAC patients with PS not reaching statistical significance may reflect limited statistical power, but given the current data, the true absence of prognostic effect cannot be excluded.

The quantitative histology-based assessment of pancreatic fat reported by Fukuda et al. [27] provided evidence that PS as defined by HPFF ≥11.3% is an independent poor prognostic indicator in multivariate analyses, with a hazard ratio of 2.32 (95% CI 1.38–3.91, *p* = 0.0015) and 2.33 (95% CI 1.42–3.81, *p* = 0.00080) for OS and PFS, respectively. 

Considering the 1/3 positive imaging studies and 2/2 positive histology studies, our results show that histology-based studies suggest an adverse prognostic role, whereas imaging-based evidence remains inconsistent.

An important source of heterogeneity across studies may relate to differences in the assessment of pancreatic fat. Histological evaluation directly quantifies adipocyte infiltration within the pancreatic parenchyma and is therefore considered a more biologically relevant measure of true fatty pancreas. In contrast, CT-based assessment relies on attenuation values as a surrogate marker of fat content and may be influenced by several factors independent of adipocyte infiltration, including pancreatic fibrosis, glandular atrophy, inflammatory changes, and technical parameters related to image acquisition [30,31,32]. Furthermore, pancreatic atrophy secondary to ductal obstruction and chronic inflammation, along with cancer-related parenchymal remodeling, may itself influence radiologic attenuation measurements, increasing the possibility of reverse causality in imaging-based studies. Consequently, histology may better capture the biological effects of ectopic fat accumulation on the tumor microenvironment, whereas CT attenuation may incompletely reflect pancreatic fat burden. This discrepancy could partly explain why histology-based studies such as Fukuda et al. [27] demonstrated a strong adverse prognostic effect of fatty pancreas, whereas CT-based studies, including those by Chan et al. [28] and Dong et al. [29], inconsistently identified an association with survival.

Overall, the methodological quality was heterogeneous. Fukuda et al. [27] demonstrated the lowest RoB, owing to quantitative histologic assessment of pancreatic fat, comprehensive follow-up, and multivariable Cox regression analyses. The remaining studies were judged to have moderate-to-high risk of bias, primarily due to their single-center designs, small sample sizes, incomplete adjustment for confounding factors, and limited multivariable prognostic modeling; see Table 2, Supplementary File S2.

## 4. Discussion

PS, also termed FP, pancreatic lipomatosis, non-alcoholic fatty pancreas disease (NAFPD) or more recently MASPD (metabolic dysfunction associated steatotic pancreatic disease), in light of the nomenclature change from MASLD, is increasingly being recognized as a significant clinical entity with impact on exocrine pancreatic diseases. While the 2026 consensus report on FP [33] states that imaging is an important diagnostic tool for diagnosing and severity assessment of FP, CT is acknowledged as being less sensitive for mild disease and MRI quantification requires the use of proton density fat fraction (PDFF) sequences. In CT, the standard evaluation for pancreatic fat is the difference between pancreatic and splenic attenuation, specifically the P/S ratio, which correlates with the histologically confirmed fat fraction [34,35]. In addition to diagnostic performance, in daily practice, we must account for the fact that though MRI provides superior accuracy, CT offers greater accessibility; this positions CT as a practical, retrospectively applicable tool for pancreatic fat assessment, particularly given the clinical ubiquity of abdominal CT in PDAC workup. 

Moreover, there are still no universally validated objective prognostic cut-off in radiological techniques for the role of PS in PDAC survival, underscoring the current gap in prognosis-specific evidence. Several epidemiological studies have used a P/S ratio below 0.70 as the cut-off for pancreatic steatosis [36], while others have proposed a threshold of 0.8 [37]. This heterogeneity of cut-offs, combined with the absence of standardized measurement protocols—whether whole-gland mean, region-of-interest sampling, or pancreas-specific software—makes cross-study comparisons unreliable. Janssens et al. attempted to address this by using a convolutional neural network to segment 19,456 images from 469 non-contrast CT scans, establishing age- and sex-specific HU distribution thresholds [38]. Additionally, Janssens et al. also demonstrated a strong correlation between whole-pancreas MR proton-density fat fraction (MR-PDFF) and CT-HU, supporting the use of both modalities for fat quantification [39]. 

The current systematic review provided very limited data on the prognostic role of PS in PDAC outcomes. While two histology-based and one CT-based assessment of PS provided insight on worse survival and increased nodal metastasis in PDAC patients with PS (Mathur 2009 [25], Mathur 2011 [26], Fukuda 2025 [27]), two other radiologically based studies provided either non-significant differences (Dong, 2025 [29]) or even opposite trends (Chan 2024 [28]). 

Despite the plausible biology behind the negative impact of PS on PDAC prognosis, with proposed mechanisms consisting in adipokine-driven inflammation, pancreatic stellate cell activation, oxidative stress, and tumor-microenvironment remodeling [40,41,42], the prognostic signal is weak and inconsistent across studies and does not support a strong adverse survival effect. A major issue is the potential confounding factors, as PS often coexists with obesity, insulin resistance, diabetes, chronic pancreatitis, and MASLD, all of which can influence both cancer biology and survival.

Among the limitations of our review and, in fact, the limitations in evaluating the prognostic role of PS for PDAC is the methodological heterogeneity between CT-based and histological approaches—while the former measures living patients pre-operatively, the latter requires resected specimens—this constitutes a fundamental divergence in this field that limits unified interpretation. Also, we must acknowledge a selection bias in the fact that reported data are from surgical cohorts.

Although CT-assessed fatty pancreas is increasingly recognized as a marker of PDAC susceptibility, the present literature does not consistently demonstrate an independent adverse effect on overall or recurrence-free survival after pancreatic cancer diagnosis; therefore, its prognostic role remains unvalidated and requires prospective multicenter confirmation. Several authors have evaluated the role of visceral fat as a surrogate for PS and analyzed its correlation with PDAC outcomes, but this is more difficult to use in daily practice as a prognostic biomarker compared to pancreatic attenuation as measured by the pancreatic-to-spleen ratio [43,44,45,46,47]; additionally, some authors have demonstrated using PET/CT that CT attenuation and FDG uptake of visceral adipose tissue are significant independent predictors of OS, suggesting that fatty tissue metabolic activity rather than mere quantity may be the relevant driver of poor prognosis; while the metabolic fat imaging approach differs fundamentally from static attenuation-only CT metrics, this is also not feasible for routine practice [48]. Among the significant limitations of current CT-based assessment of PS, there is a high need for standardized CT protocols for intrapancreatic fat quantification—currently there are at least four measurement approaches reported in the literature, (P/S ratio, P-S difference, absolute HU, AI-based whole-gland attenuation), with various thresholds. Prospective multicenter studies using a single standardized CT protocol—ideally with concurrent histological validation—are required before CT-based fatty pancreas metrics can be embedded in clinical guidelines.

Overall, despite the trend of negative prognostic signal on PDAC survival given by PS, we are far from concluding that PS is a reliable marker to predict outcomes in pancreatic cancer given the extremely sparse evidence. Moreover, we should take into account the heterogeneity of cancer biology among PDAC, which makes it more difficult to create predictions based on structural features on the non-tumoral pancreatic parenchyma only. Not least, overall nutritional status of patients and particularly sarcopenia or myosteatosis might significantly impact PDAC prognosis, so a full metabolic evaluation of these patients is warranted [49].

The overall quality of the available evidence was limited by several methodological shortcomings—the majority of the selected studies were single-center cohorts with relatively small sample sizes, increasing the risk of selection bias and limiting generalizability. Important prognostic confounders, particularly obesity, diabetes and metabolic syndrome [50,51], were inconsistently measured and adjusted for across studies. Together, these limitations contribute to a moderate-to-high risk of bias across the current literature and reduce confidence in the observed associations between PS and survival outcomes in PDAC. Consequently, the current body of evidence should be considered hypothesis-generating rather than a definitive prognostic signal of PS on PDAC outcomes, highlighting the need for larger prospective studies using standardized measures of pancreatic fat and comprehensive adjustment for metabolic confounders. Not least, we must acknowledge the use of only two bibliographic databases as a potential limitation of our work.

Given the limited and heterogeneous prognostic evidence identified in this review, pharmacological modification of pancreatic steatosis might be explored cautiously as a hypothesis-generating research strategy for improving PDAC outcomes. GLP-1 receptor agonists and SGLT2 inhibitors represent potential candidates due to their beneficial metabolic effects, which could theoretically attenuate the inflammatory and metabolic milieu associated with PS [9]. Preliminary studies in patients with type 2 diabetes have reported reductions in intrapancreatic fat following treatment with liraglutide or dapagliflozin, with the latter also associated with reductions in circulating IL-6 and TNF-α [52,53]. However, these studies were not conducted in patients with PDAC and do not provide evidence that reducing pancreatic fat improves PDAC prognosis. Future research should therefore determine whether pharmacological reduction in pancreatic fat, assessed using standardized metrics, is reproducible and accompanied by changes in relevant metabolic and inflammatory biomarkers in PDAC populations.

## 5. Conclusions

PS is a promising imaging biomarker associated with PDAC, but current data are insufficient to establish it as an independent predictor of survival or prognosis in pancreatic cancer. Despite increasing recognition of PS as a risk factor for pancreatic carcinogenesis, there is a striking lack of studies evaluating CT/MRI or histology-assessed pancreatic steatosis as an independent prognostic biomarker in pancreatic cancer. While imaging-based prognostic studies are small and retrospective, with inconsistent survival data, an adverse prognostic signal is currently stronger in histology-based studies. Overall, the currently available evidence is scarce, predominantly retrospective and methodologically heterogenous, which precludes firm conclusions to establish PS as an independent prognostic marker for PDAC survival.

## Figures and Tables

**Table 1:** Summary of studies reporting on PS impact on survival outcomes in patients with PDAC.

Study (Author, Year)	Study Design/Population	Fat Assessment	Main Finding Related to Prognosis/Survival
Mathur et al., 2009 [25]	Retrospective case–control study; 40 patients with resected pancreatic adenocarcinoma (20 node-positive vs. 20 node-negative) undergoing pancreatic resection.	Histology-based assessment of pancreatic neck margin. Pancreatic fat quantified as number of adipocytes per 5 high-power fields.	Node-positive patients had significantly greater pancreatic fat cells (46.4 vs. 21.4/5 HPF; *p* < 0.02) and lower fibrosis. Mean survival was significantly shorter in node-positive patients (18.9 vs. 30.8 months; *p* < 0.04).
Mathur et al., 2011 [26]	Retrospective cohort; 42 patients undergoing pancreatoduodenectomy for pancreatic adenocarcinoma.	CT-based assessment. Pancreatic steatosis quantified by pancreatic attenuation (HU) on non-contrast CT.	Patients with nodal metastases had significantly decreased pancreatic attenuation (higher steatosis). Node-positive patients had markedly worse survival (11 vs. 21 months; *p* < 0.01).
Chan et al., 2024 [28]	Retrospective imaging study; 101 newly diagnosed pancreatic cancer patients. CT and MRI datasets reviewed.	Mixed CT/MRI assessment. CT: pancreas attenuation lower than spleen on non-contrast CT. MRI: signal drop on out-of-phase imaging as compared to the in-phase images.	Survival higher in patients with FP (30% vs. 14.6%, *p* = 0.322)
Dong et al., 2025 [29]	Retrospective surgical cohort; 116 pancreatic cancer patients undergoing surgery.	Fatty pancreas (FP) assessed by CT imaging.	Median OS was 19.5 months in FP patients (*n* = 54) vs. 24.6 months in non-FP patients (*n* = 26), but the difference was not significant (HR 1.031, *p* > 0.05).
Fukuda et al., 2025 [27]	Retrospective cohort; 87 patients with pathologically confirmed PDAC undergoing potentially curative pancreatectomy.	Histology-based assessment. Histological pancreatic fat fraction (HPFF) measured on H&E slides from non-tumor pancreas. FP defined as HPFF ≥11.3%.	Patients with FP had significantly worse OS and RFS than those in the non-FP group: 1.34 vs. 2.71 years (*p* = 0.012) and 0.48 vs. 1.32 years (*p* = 0.00060), respectively.

Abbreviations: PDAC—pancreatic ductal adenocarcinoma, H&E—hematoxylin and eosin, OS—overall survival, RFS—recurrence-free survival, FP—fatty pancreas.

**Table 2:** Risk of bias assessment of selected studies.

Study	Participation	Attrition	Prognostic Factor Measurement	Outcome Measurement	Confounding	Analysis	Overall ROB
Mathur 2009 [25]	🟡	🟢	🟡	🟢	🔴	🟡	🔴
Mathur 2011 [26]	🟡	🟢	🟡	🟢	🔴	🟡	🔴
Chan 2024 [28]	🟡	🟢	🟡	🟢	🔴	🟡	🟡
Dong 2025 [29]	🟡	🟡	🟡	🟢	🔴	🟡	🔴
Fukuda 2025 [27]	🟢	🟢	🟢	🟢	🟡	🟢	🟡

Legend: 🟢—low risk of bias, 🟡—moderate risk of bias, 🔴—high risk of bias.

## Data Availability

No new data were created or analyzed in this study. Data sharing is not applicable to this article.

## Supplementary Materials

The following supporting information can be downloaded at https://www.mdpi.com/article/10.3390/diagnostics16152476/s1, Supplementary File S1—Search strings used for the literature search; Supplementary File S2—Risk-of-bias assessment; Supplementary File S3—PRISMA 2020 Checklist.
