# Supplementary material for: Pancreatic Steatosis and Survival Outcomes in Pancreatic Ductal Adenocarcinoma: A Systematic Review of the Current Evidence
Source: Diagnostics (Basel). 2026 Aug 6;16(15):2476. doi: 10.3390/diagnostics16152476 (PMC13464814; doi:10.3390/diagnostics16152476)
Supplement: Supplementary file 1 [file diagnostics-16-02476-s001.zip › Supplementary File S1.pdf]

Supplementary file S1

PubMed search string

("fatty pancreas"[tiab] OR "pancreatic steatosis"[tiab] OR "pancreatic fat"[tiab] OR "pancreatic fatty infiltration"[tiab] OR "fatty pancreas disease"[tiab] OR "pancreatic lipomatosis"[tiab] OR "intrapancreatic fat"[tiab] OR "pancreatic fat deposition"[tiab])
AND
("Pancreatic Neoplasms"[Mesh] OR "pancreatic cancer"[tiab] OR "pancreatic ductal adenocarcinoma"[tiab] OR PDAC[tiab] OR "pancreatic adenocarcinoma"[tiab])
AND
(prognosis[tiab] OR prognostic[tiab]OR survival[tiab] OR mortality[tiab] OR outcome*[tiab] OR recurrence[tiab] OR lethality[tiab] OR metasta*[tiab] OR dissemination[tiab] OR aggressiveness[tiab] OR "lymph node metastasis"[tiab] OR "tumor progression"[tiab])

Scopus search string

TITLE-ABS-KEY
("fatty pancreas" OR "pancreatic steatosis" OR "pancreatic fat" OR "pancreatic fatty infiltration" OR "pancreatic lipomatosis" OR "intrapancreatic fat" OR "pancreatic fat deposition")
AND
("pancreatic cancer" OR "pancreatic ductal adenocarcinoma" OR PDAC OR "pancreatic carcinoma")
AND
(prognosis OR prognostic OR survival OR mortality OR outcome* OR recurrence OR metastasis)
