# Supplementary material for: Pancreatic Steatosis and Survival Outcomes in Pancreatic Ductal Adenocarcinoma: A Systematic Review of the Current Evidence
Source: Diagnostics (Basel). 2026 Aug 6;16(15):2476. doi: 10.3390/diagnostics16152476 (PMC13464814; doi:10.3390/diagnostics16152476)
Supplement: Supplementary file 1 [file diagnostics-16-02476-s001.zip › Supplementary File S2.pdf]

Supplementary file S2

Risk of bias was assessed using the Quality in Prognosis Studies (QUIPS) tool across six domains: study participation, study attrition, prognostic factor measurement, outcome measurement, study confounding, and statistical analysis and reporting. The concise traffic-light summary presented in the main manuscript is supported below by a domain-specific justification for each judgment.

Paper	Study participation	Attrition	Prognostic factor measurement	Outcome measurement	Confounding	Analysis and reporting
Mathur et al., 2009	**Moderate risk**<br><br>Small matched case–control study, including only 40 resected PDAC patients from a database of > 1000 PD.	**Low risk**<br><br>Survival obtained from patient records and Social Security database.	**Moderate risk**<br><br>Histologic fat quantified by adipocyte counts.<br><br>Pathology reviewed by two trained investigators.<br><br>Interobserver reliability not reported.	**Low risk**<br><br>Overall survival objectively measured.	**High risk**<br><br>The inferred fat-survival relationship was indirect.<br><br>No multivariable survival analysis.	**Moderate risk**<br><br>Only univariate analysis was done.
Mathur et al., 2011	**Moderate risk**<br><br>Small retrospective surgical cohort (n=42).	**Low risk**<br><br>Survival reported for all patients.	**Moderate risk**<br><br>CT imaging measured by two blinded radiologists.	**Low risk**<br><br>Overall survival - objective endpoint.	**High risk**<br><br>No multivariable adjustment.<br><br>Nodal status strongly linked to survival.	**Moderate risk**<br><br>Kaplan–Meier curves used for survival analysis, without adjusted HR.

			Interobserver reliability not reported.			
Chan et al., 2024	**Moderate risk**<br><br>Retrospective hospital-based imaging cohort (n=101).	**Low risk**<br><br>Survival follow-up described clearly.	**Moderate risk**<br><br>Imaging interpretations made by at least two radiologists.<br><br>Mixed modalities (CT/MRI) introduce heterogeneity.	**Low risk**<br><br>Survival objectively assessed.	**High risk**<br><br>No robust multivariable prognostic model.	**Moderate risk**<br><br>Kaplan–Meier analyses used.<br><br>Limited adjustment.<br><br>Mixed CT/MRI measurement not addressed analytically.
Dong et al., 2025	**Moderate risk**<br><br>Retrospective single-center surgical cohort (n=116).	**Moderate risk**<br><br>Follow-up available in only 80/116 patients	**Moderate risk**<br><br>Objective CT definition: pancreas/spleen attenuation ratio <0.7.<br><br>Interobserver reliability not reported.	**Low risk**<br><br>Survival objectively assessed.	**High risk**<br><br>No multivariable survival adjustment reported.	**Moderate risk**<br><br>Limited prognostic modeling.
Fukuda et al., 2025	**Low risk**<br><br>Surgically treated PDAC population	**Low risk**<br><br>Follow-up complete.	**Low risk**<br><br>Blinded quantitative HPFF assessment. assessment.	**Low risk**<br><br>Survival outcomes objectively measured.	Moderate risk<br><br>Multivariable Cox analysis performed.	Low risk<br><br>Appropriate Kaplan–Meier analysis.

	(n=87). Screening flow clearly described.				Residual confounding possible for potential relevant metabolic confounders.	Multivariable Cox modeling.
